# Candidate genes associated with red colour formation revealed by comparative genomic variant analysis of red- and green-skinned fruits of Japanese apricot (*Prunus mume*)

**DOI:** 10.7717/peerj.4625

**Published:** 2018-05-04

**Authors:** Xiaopeng Ni, Song Xue, Shahid Iqbal, Wanxu Wang, Zhaojun Ni, Muhammad Khalil-ur-Rehman, Zhihong Gao

**Affiliations:** 1 Laboratory of Fruit Tree Biotechnology, College of Horticulture, Nanjing Agricultural University, Nanjing, China; 2 Jiangsu Key Laboratory for Horticultural Crop Genetic Improvement, Nanjing, China

**Keywords:** *Prunus mume*, Sequencing, Anthocyanin, UFGT, Gene expression

## Abstract

The red-skinned fruit of Japanese apricot (*Prunus mume* Sieb. et Zucc) appeals to customers due to its eye-catching pigmentation, while the mechanism related to its colour formation is still unclear. In this study, genome re-sequencing of six Japanese apricot cultivars was carried out with approximately 92.2 Gb of clean bases using next-generation sequencing. A total of 32,004 unigenes were assembled with an average of 83.1% coverage rate relative to reference genome. A wide range of genetic variation was detected, including 7,387,057 single nucleotide polymorphisms, 456,222 insertions or deletions and 129,061 structural variations in all genomes. Comparative sequencing data revealed that 13 candidate genes were involved in biosynthesis of anthocyanin. Significantly higher expression patterns were observed in genes encoding three anthocyanin synthesis structural genes (*4CL*, *F3H* and *UFGT*), five transcription factors (MYB–bHLH–WD40 complexes and NAC) and five anthocyanin accumulation related genes (*GST1*, *RT1*, *UGT85A2*, ABC and MATE transporters) in red-skinned than in green-skinned Japanese apricots using reverse transcription-quantitative polymerase chain reaction. Eight main kinds of anthocyanin s were detected by UPLC/MS, and cyanidin 3-glucoside was identified as the major anthocyanin (124.2 mg/kg) in red-skinned cultivars. The activity of UDP-glucose flavonoid-3-*O*-glycosyltransferase enzyme determined by UPLC was significantly higher in all red-skinned cultivars, suggesting that it is the potential vital regulatory gene for biosynthesis of anthocyanin in Japanese apricot.

## Introduction

Japanese apricot (*Prunus mume* Sieb. et Zucc), an attractive fruit tree, originated from Southwest China and has been extensively cultivated in all of East Asia and Japan. China, being the origin of Japanese apricot, is rich in good quality germplasm with an approximately 190 fruiting cultivars (Chu, 1999). The fruit is usually processed into many value-added products, containing salted-fruit, drinks and juice, is well advised to high nutritional and medicinal value (Chen, 1989). Fruit colour is an significant factor for determining fruit quality, which increases its value and attracts consumer’s attention (Gao, Han & Zhang, 2003). The whole genome of Japanese apricot was sequenced in 2012 (Zhang et al., 2012), laying the foundation for detailed study of important traits related to pigmentation, flowering time, dormancy and other commercially relevant aspects. Moreover, genome-based tools can also be advanced to develop breeding efficiency and carry out other functional genomic research.

The emergence of next-generation sequencing (NGS) technologies had a tremendous impact on genomic research over the past decade. NGS technologies have become a major tool for acquiring data related to genetic variation inside a variant and recognizing exclusive genotypic molecular markers. Sequencing technologies have been applied to discover and quantify genomic variants in many species of plants and enabled researchers to illustrate genomic alterations among specific genotypes (Zhang et al., 2014). Sequencing-based bulked segregation analysis (seq-BSA) was used to map resistance genes by re-sequencing in pigeon pea and has been proven an effective way to carry out genomics-assisted breeding. Whole genome re-sequencing has also been helpful in identifying the particular location of equally crossover and non-crossover recombination measures in an Avian pedigree (Smeds et al., 2016). Whole genome re-sequencing has made genomic research easier and more accurate.

Anthocyanins are the main water-soluble pigments localized in vacuoles belonging to parent class of flavonoids synthesized via the phenylpropanoid pathway and accumulated in many flowers, fruits, leaves, stems, roots and other plant tissues (Mol, Grotewold & Koes, 1998; Dixon & Steele, 1999; Escribano-Bailón, Santos-Buelga & Rivas-Gonzalo, 2004; Espley et al., 2007; Welch, Wu & Simon, 2008; Jimenez-Garcia et al., 2013). The accumulation of anthocyanins contributes to diverse characteristic: red, purple or blue hues, reliant on pH of the vacuole, which not only attracts consumers’ attention but also contributes to fruit quality and its medicinal importance (Rao & Rao, 2007; Singh et al., 2008; Wang & Stoner, 2008; He & Giusti, 2010; Batra & Sharma, 2013).

The anthocyanin biosynthesis pathway has been widely reported in most fruits: apple (Kim et al., 2003), grapes (Boss, Davies & Robinson, 1996a), peach (Tsuda et al., 2004), pear (Steyn et al., 2004) and plum (Usenik, Štampar & Veberič, 2009). Primary structural pathway genes encoding enzymes and transcription factors (TFs), which regulate structural gene transcription, are two main elements involved in regulation of anthocyanin synthesis (Koes, Verweij & Quattrocchio, 2005). The specific flavonoid pathway involving several structural genes and the phenylpropanoid pathway was the first pathway discovered, in which cinnamic acid 4-hydroxylase (*C4H*), phenylalanine ammonia-lyase (*PAL*) and 4-coumarate-CoA ligase (*4CL*) converted phenylalanine to coumarate-CoA. We found some structural genes in the flavonoid pathway which named chalcone isomerase (*CHI*), chalcone synthase (*CHS)*, dihydroflavonol 4-reductase (*DFR*), flavanone 3-hydroxylase (*F3H*), flavonol 3′-hydroxylase (*F3′ H*), flavonol synthase (*FLS*), leucoanthocyanidin dioxygenase (*LDOX*) and UDP-glucose flavonoid-3-*O*-glycosyltransferase (*UFGT*). Expression of structural genes in pathways could be regulated directly by TFs whose functions have been demonstrated in many plants. An intricate of MYB TF, basic helix-loop-helix (bHLH) TFs and WD-repeat proteins in MYB–bHLH–WD40 (MBW) complex combined with promoters of structural genes in anthocyanin biosynthesis (Gonzalez et al., 2008). In apple and pear, expression of the *MYB10* gene has been shown to control red apple fruit flesh and foliage anthocyanin accumulation to the exclusion of other non-red pigments (Takos et al., 2006; Espley et al., 2007). The biosynthesis of anthocyanins in lotus (*Nelumbo* Adans) was regulated by MBW complex (*NnMYB5*, *NnbHLH1* and *NnTTG1*); the study showed that *NnMYB5* was a transcriptional activator of anthocyanin synthesis that interacted with *NnbHLH1* and *NnTTG1* (Sun et al., 2016). TTG1, a WD40 protein, participated in anthocyanin synthesis had also been characterized from fruit species including grapevine (Kobayashi, Goto-Yamamoto & Hirochika, 2004) and strawberry (Schaart et al., 2013).

In Japanese apricot, fruit colour is ascertained by the accumulation of anthocyanins, most abundant flavonoids. Cyanidin 3-glucoside remained the major anthocyanin in several diversities of red-skinned cultivars. In this present study, red-skinned cultivars were found to be more attractive to consumers than their green counterparts due to their eye-catching appeal and therefore were used for more findings of anthocyanin biosynthesis.

## Materials and Methods

### Plant materials and DNA extraction

Fruits of six different cultivars of Japanese apricot were collected from National Field Genebank for *P. mume* and Waxberry at the Jiangpu Agricultural Research Station, Nanjing Agricultural University, Nanjing, P.R. China. Based on fruit skin colour, these cultivars were classified into two groups: green- and red-skinned cultivars. Green-skinned cultivars included ‘QingjiaNo.2’ (QJM), ‘Yanglao’ (YLM) and ‘Shinano koume’ (SKM), and red-skinned cultivars included ‘Ruantiao hongmei’ (RHM), ‘Xiaoye zhugan’ (XZM) and ‘Zhonghong’ (ZHM). Red-skinned cultivars can be easily distinguished from green-skinned by their specific phenotypic traits. DNA samples were extracted from young leaves of all cultivars using CTAB method following the manufacturer’s protocol to perform genome re-sequencing. Fruits were harvested in triplicate for re-sequencing on 28th May and 2nd June.

### Exploration of physiological indicators and phenotypic traits

**Figure 1:**
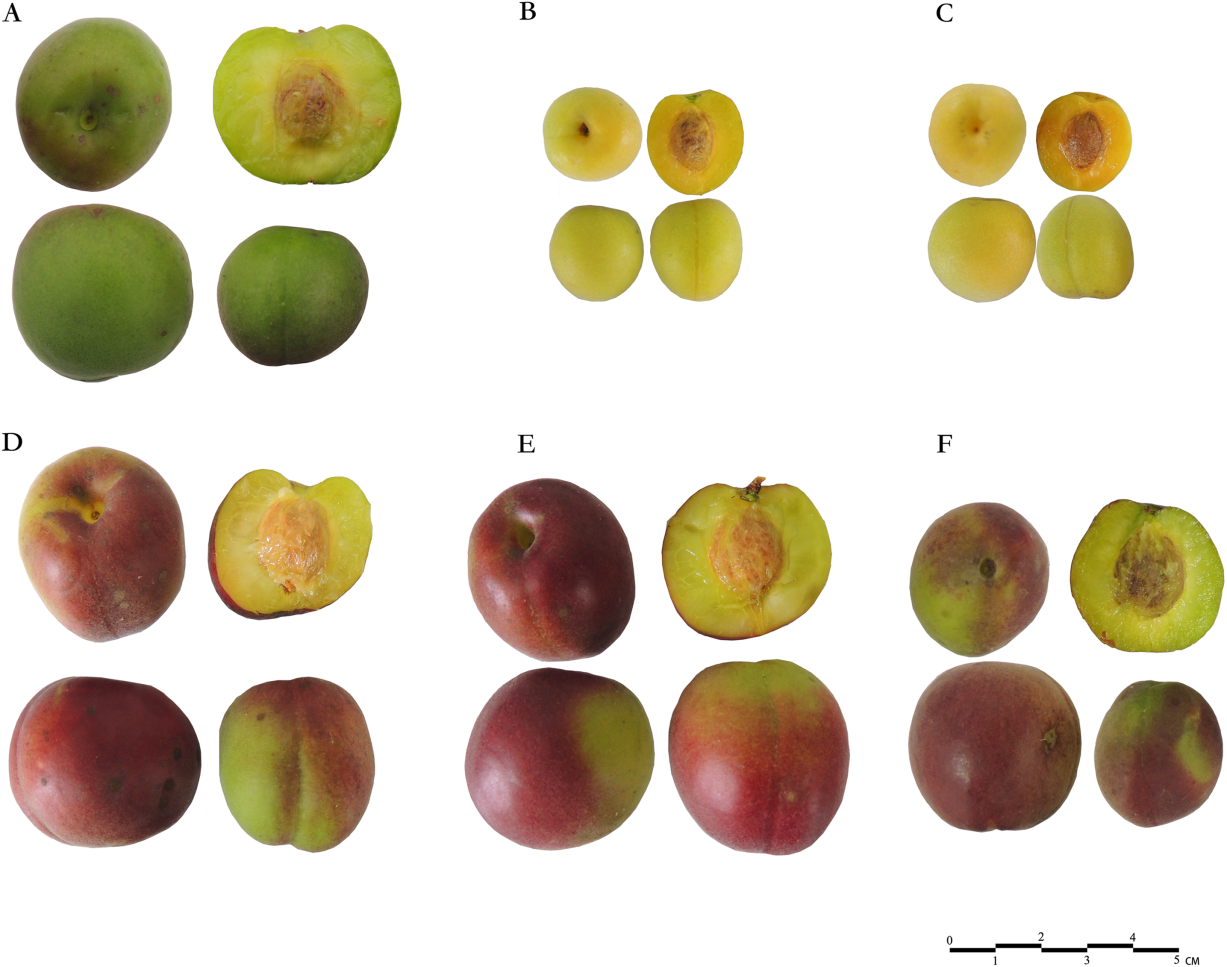
Fruits of six Japanese apricot cultivars used in this study. The top row are three green-skinned cultivars: (A) ‘QJM,’ (B) ‘SKM,’ (C) ‘YLM’; the lower row are three red-skinned cultivars: (D) ‘RHM,’ (E) ‘XZM,’ (F) ‘ZHM.’ The photographs are provided by Xiaopeng Ni.

Physiological indicators were measured at the same ripening time (Fig. 1). The vertical height, flank diameter and width were measured by vernier calliper (GuangLu, Guilin, China). Single fruit weight was measured with an electronic balance (METTLER TOLEDO, Zurich, Switzerland). Fruits were peeled and pulp was squeezed in a cheesecloth, and filtered, homogenized juice was used for determination of soluble solids contents using a digital refractometer (ATAGO, Tokyo, Japan).

The colour of the fruits was observed and calculated with a Minolta CR-400 Chroma Meter (Konica Minolta Sensing, Inc., Osaka, Japan) using an illumination of D75 and at measuring angle of 10° after calibration with an ordinary white plate (*Y* = 94.00, *x* = 0.3158, *y* = 0.3322). Three records of L* represents lightness, a* and b* represent red–green and yellow–blue chromaticity which coordinates were noted for individually Japanese apricot sunny surface. Each sample was chosen from 10 replicates by changing the position of sunny surface at maturity stage. Chroma and hue angle were calculated as Chroma = (a*^2^ + b*^2^)^1/2^ and Hue = tan^−1^(b*/a*) (Oms-Oliu, Soliva-Fortuny & Martín-Belloso, 2008).

### Construction of libraries and Illumina sequencing

To construct a whole genome shotgun sequencing library for whole genome re-sequencing, high-quality genomic DNA was extracted and randomly interrupted by ultrasonic shearing at intervals of 150–800 bp (pair-end library). DNA fragments of required length were isolated by electrophoresis, and then Klenow DNA polymerase, T4 DNA polymerase and T4 PNK were utilized to convert sticky ends to blunt ends. Amplified DNA fragments were finally sequenced using an Illumina HiSeq™ 2500 platform (1gene, Zhejiang, China). The sequences were aligned to the Japanese apricot reference genome (https://doi.org/10.6084/m9.figshare.6197219.v1) using the SOAP2 (Short Oligonucleotide Alignment Program 2) software (http://soap.genomics.org.cn/soapaligner.html), and SOAP2 was matched to the single-end and paired-end fragment. According to alignment results, it was possible to calculate the depth and coverage of sequencing with respect to reference genome. Statistical analyses were carried out by means of SPSS 18.0 software (SPSS Inc., Chicago, IL, USA).

### Single nucleotide polymorphisms detection

Single nucleotide polymorphisms (SNPs) were recognized using SOAPsnp (http://soap.genomics.org.cn/soapsnp.html) to explain the effects of substitutions (including synonymous SNPs and non-synonymous SNPs). According to the comparison results, a Bayesian model was used to determine the probability of each possible genotype based on actual observed data, while considering data characteristics, sequencing quality and experimental factors. The genotype of specific locus of sequenced individual was selected as the maximum of probability and a quality value reflecting genotype was assigned on the basis of that probability, then consensus sequence was obtained. Based on this consensus sequence, reference sequence exists, polymorphism screening and filtering was done. The SAM tools function ‘mpileup’ was used to identify unprocessed SNPs population by means of reads through a mapping value ≥20. Using the SAM tools database ‘vcfutils,’ SNPs extracted using the beyond procedure were initially filtered to yield sequencing depths between 35 and 91. Raw SNPs sites were additionally filtered based on subsequent criteria: copy number ≤2, and a least of 5 bp apart, with the exclusion of insignificant allele frequencies (MAF ≥ 0.05) where SNPs were engaged when distance between SNPs was <5 bp. To check the SNPs calling accuracy of SAMtools, fragments were casually selected and amplified using corresponded primers, and subsequent PCR products exposed to Sanger sequencing for concordance rates. Both HTML and text production files were created from SOAPsnp; output included the chromosome (Chr), position, reference base <-> sample base, SNPs status, strand, annotation type, feature type, codon phase (phase of the codon mutation), codon mutate (the codon before mutation <-> the codon after mutation), amino acid mutate (amino acids before mutation <-> amino acids after mutation), synonymous mutations, non-synonymous mutations, start position, end position, gene id and its functions. Using these annotation files, SNPs could correspond to a homologous functional element region, such as coding sequence (CDS) region, exon region, UTR region and others. This information could help us to select desired SNPs.

### Insertions or deletions detection

SOAPindel software was designed for all paired-end sequencing data, wherein SOAPindel comparison allowed open gaps, and short insertions or deletion (InDel) detections was performed. During short InDels testing, all reads met paired-end requirements, and only one end gaps contained the alignment. In this process, the length of gap was set, not more than 5 bp. In the detection of InDels, at least three pairs of reads were required to define an InDel. For InDels annotation, analysis of regional information and impact of InDels on CDS region, such as in a frameshift mutation, was possible.

### Structural variations detection

The SOAPsv (http://soap.genomics.org.cn/SOAPsv.html) program was used to detect structural variations by whole genome de novo assembly system requirements. Structural variations (SVs) is one of the important variations between individuals of the same species. In contrast to paired-end, if there is a structural difference an individual sequence and the reference sequence, a comparison cannot be made. The abnormal condition of this double end alignment can be used to detect SVs. At present, the main types of SVs can be detected, such as insertion, deletion, duplication (tandem duplication and dispersed duplication), inversion (upstream and downstream) and complex. Sometimes, two types of SVs can occur simultaneously.

### DNA level functional annotation and screening of variant genes

Gene functions were annotated by BLAST alignment with Japanese apricot reference genes (Zhang et al., 2012) (http://www.ncbi.nlm.nih.gov/genome/?term=Prunus+mume) and *Arabidopsis* proteins (Huala et al., 2001) (http://www.arabidopsis.org/). In order to analyse the gene’s biological function, we mapped all the different genes to NR (non-redundant sequence databases) (Holm & Sander, 1998) (http://www.ncbi.nlm.nih.gov/refseq/about/nonredundantproteins/), Swiss-Prot protein database (Swiss-Prot) (Bairoch & Apweiler, 2000) (http://web.expasy.org/docs/swiss-prot_guideline.html), GO (Gene Ontology) (Ashburner et al., 2000) (http://geneontology.org/page/go-database), COG (Clusters of Orthologous Groups) (Tatusov, Koonin & Lipman, 1997) (https://www.ncbi.nlm.nih.gov/COG/) and KEGG (Kyoto Encyclopedia of Genes and Genomes) (Kanehisa et al., 2004) (http://www.genome.jp/kegg/). These annotation databases were used for comparisons with homologous genes.

Explanation of data based on homologous genes was utilized to monitor for unique genes associated with colour formation. Non-synonymous SNPs, InDels or SVs were examined on the basis of green-skinned (‘QJM,’ ‘YLM’ and ‘SKM’) and red-skinned (‘RHM,’ ‘XZM’ and ‘ZHM’) cultivars phenotypes.

### Reverse transcription-quantitative polymerase chain reaction

Reverse transcription-quantitative polymerase chain reaction (qRT-PCR) was carried out to identify the comparative alteration in expression of genes identified in re-sequencing analysis. Total RNA samples were extracted from peels using CTAB extraction method and used for expression analysis (Tong et al., 2009).

SuperScript II RT (Invitrogen) was used to synthesize the first-stand cDNA from Individual sample total RNA with an Oligo primer. qRT-PCR reactions were performed in 96-wells plates using a 7300 Real-Time PCR System (Applied Biosystems, Foster City, CA, USA) using SYBR Green PCR Master Mix (Applied Biosystems, Foster City, CA, USA). Beacon designer 7.0 program (Premier Biosoft International, Palo Alto, CA, USA) was used to design gene-specific primers. Each qRT-PCR reaction (20 μL) confined 8.6 μL ddH_2_O, 0.2 μL (100 nM) of each primer, 10 μL SYBR Green II Master Mix and 1 μL of diluted cDNA. To standardize the entire quantity of cDNA in individually reaction, a housekeeping gene, *RPII* (sense: 5′-TGAAGCATACACCTATGATGATGAAG-3′, antisense: 5′-CTTTGACAGCACCAGTAGATTCC-3′) was used as a reference gene in control reactions (Tong et al., 2009). All primers that were used in reactions are listed in Table 1. The expression levels of relative genes were measured using the 2^−ΔΔCt^ method (Ramakers et al., 2003).

**Table 1:** Sequences of gene associated the biosynthesis of anthocyanin primers used in qRT-PCR analysis of different genes.

Functions	Gene	Primer name	Sequence 5′-3′	Gene ID	Size (bp)
Structural genes	*4-CL*	4-CL.F	CGCCAAAATCCTTCAACCC	103339831	298
		4-CL.R	ACGATTGAGAAGAAGCAGAC		
	*F3H*	F3H.F	CCAAGTGGCAACTGCTGAAAC	103326444	413
		F3H.R	CCGTGATCTCCCTTGCTCAG		
	*UFGT*	UFGT.F	ATTAAATGCCTTTGAATTGGTA	103328988	297
		UFGT.R	CAAAATCAGAACCACCGAAT		
Transcription factors	*MYB114*	MYB114.F	GTAGACTAAGGTGGTTGAA	103342473	139
		MYB114.R	TGATGGTTAGGGAAGAGA		
	*MYB29*	MYB29.F	GCAGCTTGACCATCCTCCATA	103334039	307
		MYB29.R	ATGGTGGGTTGTTGGGTACAT		
	WD40	WD40.F	TCCGGCCTTCTCCACCGA	103323546	300
		WD40.R	ACTGCAAAGTCCCAATAGCGAAG		
	bHLH30	BHLH30.F	CTCTTCGACCCTTTTACGC	103324936	178
		BHLH30.R	GATTCTTCAAAGCCGCCAA		
	NAC	NAC.F	CCCCAAAACAGCAAAGAACGAG	103323499	667
		NAC.R	TCCCGAAAGAGCCCAACCTCA		
Regulative genes	*GST1*	GST1.F	GAAGATCCCAGTGCTCGTTC	103323824	154
		GST1.R	ATCGTCAGCGAATTTAACCC		
	*RT1*	RT1.F	CTGCCTTGCTTGTATGACCA	103335106	109
		RT1.R	CCAATGATTTCCTGCTAAACGA		
	*UGT85A2*	UGT85A2.F	TTCCAGGAATGAACGGCATC	103323808	161
		UGT85A2.R	AACATCTTGCTCCAATGCATC		
	*ABCG12*	ABCG12.F	TGGCAGAACTGTAATCACC	103326861	121
		ABCG12.R	TAGAGCCATATTTGCTTGTCC		
	*MATE1*	MATE1.F	ATCTATATGAGTCTTCGAGCA	103326834	101
		MATE1.R	TTGAGTAAACCAGCGTCCA		

**Note:**

*4-CL*, 4-coumarate-CoA ligase-like 7; *F3H*, flavanone 3-hydroxylase; *UFGT*, UDP-glucose flavonoid 3-*O*-glucosyltransferase 3-like; *MYB114*, transcription factor MYB114; *MYB29*, transcription factor MYB29-like; WD40, WD repeat-containing protein 75; bHLH30, transcription factor bHLH30-like; NAC, NAC transcription factor 29; *GST1*, probable glutathione S-transferase; *RT1*, UDP-rhamnose: rhamnosyltransferase 1; *UGT85A2*, UDP-glycosyltransferase 85A2-like; *ABCG12*, ABC transporter G family member 12-like; *MATE1*, MATE efflux family protein 1.

### Extraction and analysis of anthocyanin

Extraction of anthocyanins was carried out conferring to previously reported methods with some alterations (Pomar, Novo & Masa, 2005). Peels (2 g) were detached from pulp to obtain juice with 2% formic acid in methanol (10 mL) for 24 h in dark. The process was reiterated four times. The extracts attained were assorted and centrifuged at 12,000 rpm for 25 min, and then the supernatant was evaporated to dryness at 40 °C and reconstituted in 2% formic acid in methanol (5 mL). Individual sample was accomplished for third times, and the extracts were used for anthocyanin pigmentation identification. Cyanidin 3-glucoside (Cy-3-glc) (Sigma Chemical, St. Louis, MO, USA) was used as a standardized, dissolved in acidified MeOH (2% formic acid) to obtain a concentration of 1 mg/mL. The UPLC system with the Xevo® G2-XS QTof Mass Spectrometer was used for separation according to the method described by Hosseinian, Li & Beta (2008).

### UFGT enzyme activity

Pericarp (1 g) was pulverized with a mortar and pestle in liquid N_2_. Reaction substrate was prepared following the method (Wu et al., 2017) with some modifications. Reaction tubes including enzyme were incubated at 25 °C for 50 min, and stopped through accumulation of 20% trichloroacetic acid in methanol (75 μL).

An ultra-performance liquid chromatograph (UPLC) was fortified with a opposite segment column ACQUITY UPLC HSS C18 (1.8 μm particle sizes, 100 mm × 2.1 mm I.D.) (Waters, Milford, MA, USA) for separation. The UPLC technique was carried out with the method described by Wu et al. (2017). UFGT were enumerated using quercetin-3-galactoside (Sigma Chemical, St. Louis, MO, USA) as an ordinary. Lastly, UFGT action was characterized by mg quercetin-3-gal·g^−1^ FW.

### Statistical analysis

Analysis of variance (ANOVA) was carried out to compare cultivar mean values using IBM SPSS Statistics 18 (SPSS Inc., Chicago, IL, USA). The least significant difference test was employed to determine variances between means at a 5% significance level. GraphPad Prism version 6.0 (GraphPad Software, San Diego, CA, USA) was used for graph plotting.

## Results

### Variation in phenotypic traits of six Japanese apricot cultivars

Phenotypic observations showed a significant difference between red- and green-skinned cultivars due to red pigmentation of fruit skin (Fig. 1). ‘XZM’ showed highest red pigmentation from all cultivars followed by ‘RHM’ and ‘ZHM,’ whereas green-skinned cultivars ‘SKM’ and ‘YLM’ had trace amounts or no colour pigmentation. Phenotypic indicators revealed no significant differences in flank diameter, vertical diameter and width among all red-skinned, whereas the values for ‘QJM’ and other two green-fleshed cultivars, ‘SKM’ and ‘YLM,’ were significantly lower than those of all other cultivars (Table 2). The ‘QJM’ cultivar had the highest fruit weights, while other two green-skinned cultivars were approximately 1/8 to 1/7 the weight of ‘QJM.’ The recorded fruit weights of three green-skinned cultivars ‘QJM,’ ‘SKM’ and ‘YLM’ were 36.89, 5.27 and 4.36 g, respectively. The vertical diameter of all red-skinned were more than all green-skinned cultivars, for ‘width and flank diameter,’ ‘QJM’ and all red-skinned cultivars were close in value and had no significant difference except two green-skinned cultivars, ‘SKM’ and ‘YLM.’ The weights of red-skinned cultivars ‘RHM,’ ‘XZM’ and ‘ZHM’ were recorded as 26.68, 24.22 and 30.17 g, respectively (Table 2). The highest soluble solids were found in ‘QJM’ (9.16%), closely matched by ‘SKM’ (9.12%), whereas the concentration in other cultivars ranged between 5.79% and 8.83%. The soluble solids contents in red-skinned cultivars were recorded lower than those in green-skinned cultivars (Table 2).

**Table 2:** Skin colour indexes of six Japanese apricot cultivars.

Parameters	Green-skinned			Red-skinned		
	‘QJM’	‘SKM’	‘YLM’	‘RHM’	‘XZM’	‘ZHM’
Vertical diameter (mm)	33.04 ± 0.83^c^	22.31 ± 0.38^d^	20.11 ± 0.38^e^	34.37 ± 0.49^b^^c^	35.21 ± 0.49^b^	37.59 ± 1.03^a^
Width (mm)	35.95 ± 0.85^b^	20.61 ± 0.32^c^	19.58 ± 0.35^c^	38.42 ± 0.55^a^	35.82 ± 0.55^b^	37.79 ± 1.08^a^
Flank diameter (mm)	33.84 ± 1.04^a^^b^	19.76 ± 0.32^c^	18.43 ± 0.37^c^	33.79 ± 0.48^a^^b^	32.13 ± 0.52^b^	35.06 ± 1.27^a^
Per Fruit Weight (g)	36.89 ± 2.04^a^^b^	5.27 ± 0.21^d^	4.37 ± 0.25^d^	26.68 ± 0.79^b^^c^	24.22 ± 1.03^c^	30.17 ± 3.06^b^
Soluble Solids (%)	9.16 ± 0.41^a^	9.12 ± 0.33^a^	8.22 ± 0.22^b^	7.09 ± 0.14^c^	5.79 ± 0.21^d^	8.83 ± 0.64^b^
L*	53.76 ± 0.41^b^	59.95 ± 0.61^a^	60.41 ± 0.59^a^	30.49 ± 0.65^d^	31.52 ± 0.34^d^	33.12 ± 0.33^c^
a*	−18.83 ± 0.23^d^	−9.77 ± 1.07^c^	−17.01 ± 0.79^d^	15.93 ± 0.75^a^	16.58 ± 0.32^a^	12.62 ± 0.49^b^
b*	34.26 ± 0.31^c^	43.91 ± 0.73^a^	40.52 ± 0.84^b^	11.93 ± 0.59^d^^e^	11.18 ± 0.37^e^	13.37 ± 0.43^d^
C	39.1 ± 0.37^b^	45.35 ± 0.76^a^	44.25 ± 0.64^a^	20.08 ± 0.81^c^	20.04 ± 0.42^c^	18.53 ± 0.47^c^
h	118.78 ± 0.15^a^	101.87 ± 1.56^c^	113.15 ± 1.17^b^	37.13 ± 1.48^e^	33.82 ± 0.69^f^	46.84 ± 1.42^d^

**Note:**

L* means lightness, a* means red–green chromaticity, b* means yellow–blue chromaticity. Each sample chose from 10 replications by changing the position of the sunny surface at maturity stage. C means Chroma, and h means hue angle, they were calculated being Chroma = (a^*2^+b^*2^)^1/2^ and Hue = tan^−1^(b^*^/a^*^), respectively. Values represent as a mean ± standard deviation, *n* = 3. Different letters in rows indicate significant differences among mean values of treatments (*p* < 0.005).

CIELab parameters were also measured for these cultivars (Table 2). Significant differences appeared between red- and green-skinned cultivars. ‘XZM’ exhibited highest a* (16.58) value and lowest b* (11.18) value, representing the highest amount of red pigmentation among the studied cultivars, followed by ‘RHM’ and ‘ZHM’ with a* values of 15.93 and 12.62, respectively, and b* values of 11.93 and 13.37, respectively. ‘QJM’ showed the lowest a* (−18.83) value and a higher b* (34.26) value, revealing the highest amount of green pigmentation, whereas ‘YLM’ revealed the second lowest a* (−17.01) value above ‘SKM’ (−9.77) and a lower b* (40.52) than ‘SKM’ (43.91), indicating higher amount of green pigmentation but a lower amount of yellow pigmentation. Moreover, lightness (L*), hue angle (H*) and Chroma (C*) varied significantly between red- and green-skinned cultivars, all three parameters were higher in green-skinned than in red-skinned cultivars.

### Anthocyanin identified in Japanese apricot by UPLC/MS

**Figure 2:**
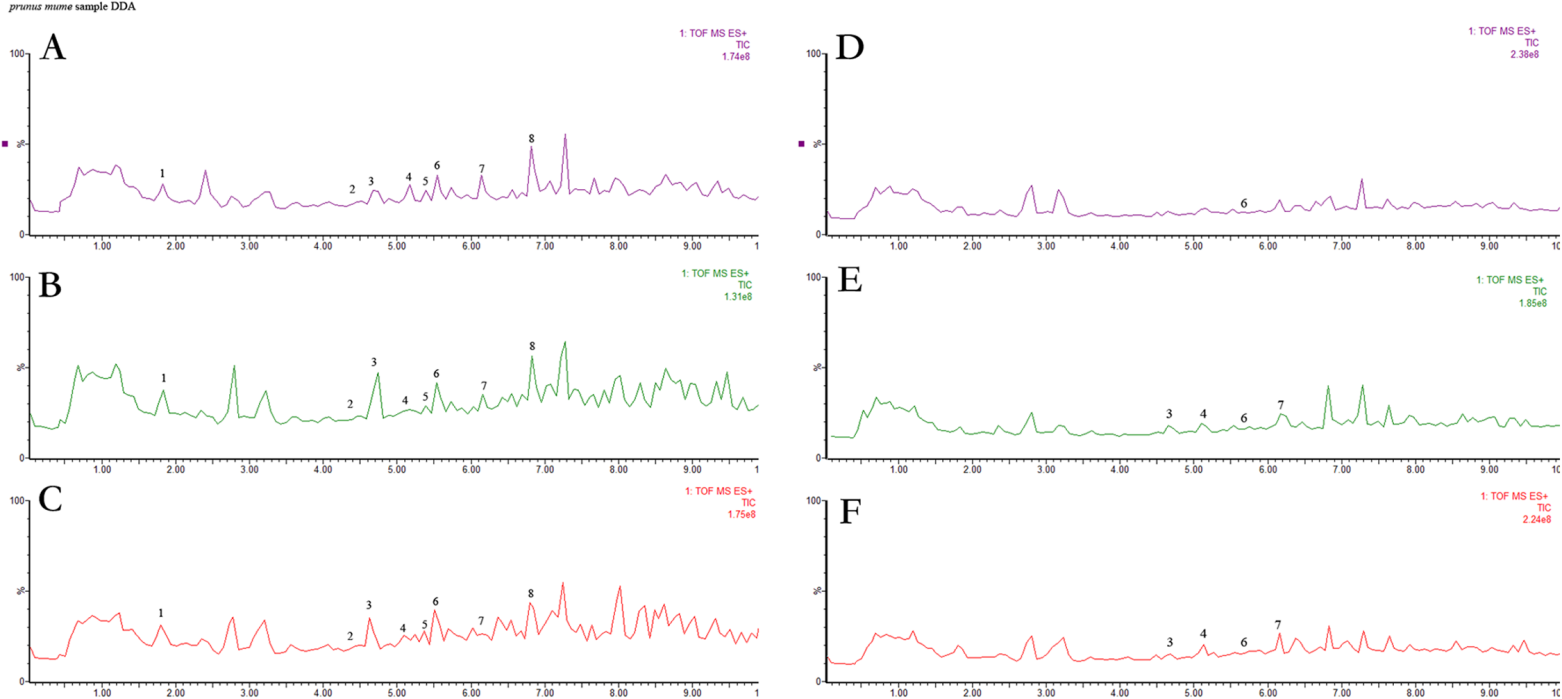
UPLC chromatograms of anthocyanin extracts captured at 520 nm for anthocyanin of six cultivars. (A) ‘ZHM,’ (B) ‘XZM,’ (C) ‘RHM,’ (D) ‘YLM,’ (E) ‘SKM,’ (F) ‘QJM.’ Peak identification is shown in Table 3.

Anthocyanins are the key pigments responsible for colour in many flowers and fruits, accumulating only during plant ripening (Lancaster et al., 1994; Salvatierra et al., 2010; Rahim, Busatto & Trainotti, 2014; Wei et al., 2015; Wang et al., 2015). Anthocyanins coordinate to the flavonoid class of compounds and structurally comprise an anthocyanidin aglycone bound to one or more sugar moieties (Jaakola, 2013). An entire of eight anthocyanin compounds were recognized on the basis of reference cyanidin-3-*O*-glusodides and mass spectrometry (MS) (Mazza, Cacace & Kay, 2004) (Fig. 2). UPLC in combination with MS provided a sophisticated technique for anthocyanin analysis. Eight anthocyanin compounds were present in all red varieties studied with differences in retention time (Rt), including cyanidin-3-*O*-glucoside (peak 1), cyanidin-3-*O*-(6-*O*-malonyl-b-d-glucoside) (peak 2), cyanidin-3,5-*O*-diglucoside (peak 3), delphinidin-3-*O*-arabinoside (peak 4), cyanidin-3-*O*-(6″-acetyl-galactoside) (peak 5), delphinidin-3-*O*-glucoside (peak 6), delphinidin-3-*O*-(6″-acetyl-galactoside) (peak 7) and peonidin-3-*O*-glucoside (peak 8) (Fig. 2), whereas few green-skinned cultivars contained all the anthocyanins. Three anthocyanin compounds: cyanidin-3,5-*O*-diglucoside, delphinidin-3-*O*-arabinoside and delphinidin-3-*O*-(6″-acetyl-galactoside) were only detected in ‘QJM’ and ‘SKM’ and not the ‘YLM’ cultivar. Delphinidin-3-*O*-(6″-acetyl-galactoside) was detected in all three green-skinned cultivars (Table 3). Cyanidin-3-*O*-glucoside (Cy-3-glu) content was the highest among all anthocyanin compounds, followed by cyanidin-3-*O*-(6-*O*-malonyl-b-d-glucoside) (Cy-3-mal-glu) and delphinidin-3-*O*-arabinoside (Dp-3-ara). Peonidin-3-*O*-Glucoside is the lowest from all anthocyanins. ‘ZYM’ had the highest content of ‘Cy-3-glu’ (124.2 mg/kg), followed by ‘RHM’ (35.2 mg/kg) and ‘ZHM’ (32.3 mg/kg) (Table 3).

**Table 3:** Anthocyanins identification in all six Japanese apricot cultivars by UPLC/MS.

Peak No.	Anthocyanins	Retention time (min)	[M]+ (m/z)	Frag.MS2 (m/z)	Content (mg/kg)					
					QJM	SKM	YLM	RHM	ZYM	ZHM
1	Cyanidin-3-*O*-glucoside	1.80	449	287	ND	ND	ND	35.2 ± 0.6^b^	124.2 ± 0.1^a^	32.3 ± 0.6^b^
2	Cyanidin-3-*O*-(6-*O*-malonyl-b-d-glucoside)	4.51	535	287	ND	ND	ND	52.8 ± 0.3^b^	99.2 ± 0.7^a^	56.2 ± 0.3^b^
3	Cyanidin-3,5-*O*-diglucoside	4.69	611	287	4.8 ± 0.5^d^	8.0 ± 0.3^c^	ND	48.8 ± 0.4^a^	24.3 ± 0.3^b^	12.8 ± 0.5^c^
4	Delphinidin-3-*O*-arabinoside	5.19	465	303	13.6 ± 0.8^c^	0.8 ± 0.6^d^	ND	72.8 ± 0.3^a^	49.6 ± 0.6^b^	88.2 ± 1.0^a^
5	Cyanidin-3-*O*-(6″-acetyl-galactoside)	5.39	491	287	ND	ND	ND	19.3 ± 0.1^a^	9.2 ± 0.8^b^	3.7 ± 0.1^c^
6	Delphinidin-3-*O*-glucoside	5.63	465	303	2.6 ± 0.3^d^	6.4 ± 0.8^c^	5.6 ± 0.3^c^	17.5 ± 0.5^a^	14.1 ± 0.4^b^	16.6 ± 0.3^a^
7	Delphinidin-3-*O*-(6″-acetyl-galactoside)	6.18	507	303	1.6 ± 0.2^c^	3.2 ± 0.4^c^	ND	10.7 ± 0.6^a^	10.4 ± 0.2^a^	5.7 ± 0.4^b^
8	Peonidin-3-*O*-Glucoside	6.99	463	301	ND	ND	ND	0.8 ± 1.0^b^	0.3 ± 0.5^c^	3.2 ± 0.4^a^

**Notes:**

Values represent as a mean ± standard deviation, *n* = 3. Different letters in rows indicate significant differences among mean values of treatments (*p* < 0.005).

ND, no detected.

### Re-sequencing of six cultivars

Genomic variants amongst the six genotypes incorporated SNPs, InDels and SVs. Six DNA libraries were built in order to perform whole genome re-sequencing. In total, 92.2 gigabits (Gb) of crude sequence data, about 15.3 Gb (clean data) per individual remained after filtering the paired-end sequences of individual cultivar (Table 4), that translated to an average of 49.1× depth of coverage per genotype. The reference genome was available from NCBI Sequence Read Archive (SRA) for alignment (BioProject PRJNA371370, accessions SRR5241550–SRR5241555), and full assembly and analysis data are available at https://doi.org/10.6084/m9.figshare.6197219.v1. Every individual sample read sequence was compared to the reference genome by means of SOAP2 (Short Oligonucleotide Alignment Program 2). The coverage rate ranged from 79.9% to 85.3%. Of all cultivars studied, ‘QJM’ had the highest coverage rate (85.3%), while ‘XZM’ (79.9%) had the lowest, and that of the other four cultivars was approximately 83%. The base mapping rate ranged from 61.3% for ‘XZM’ to 79.3% for ‘RHM,’ and 70.7% was mapped to reference sequence on average, including 55.6% that mapped uniquely.

**Table 4:** Summary of genome re-sequencing and variations for all six Japanese apricot cultivars.

Classification	Genotype	Clean bases (Gb)^a^	Coverage rate (%)^b^	Sequencing depth^c^	Mapping bases rate (%)^d^	SNP (M)^e^	InDel (K)^f^	SV (K)^g^
Green-skinned	‘QJM’	8.9	85.3	32.4	78.9	1.4	77.4	40.1
	‘SKM’	20.5	83.0	64.0	67.9	1.2	82.1	22.0
	‘YLM’	21.9	82.8	69.2	68.6	1.3	77.4	12.9
Red-skinned	‘RHM’	8.6	84.5	31.3	79.3	1.2	72.2	19.9
	‘XZM’	11.9	79.9	33.7	61.3	0.9	68.1	11.4
	‘ZHM’	20.4	83.0	63.8	68.1	1.3	79.2	22.9
	Total	92.2	83.1	49.1	70.7	7.4	456.2	129.1

**Notes:**

M and K mean million and thousand, respectively. Three total terms of last row (coverage rate, sequencing depth and mapping bases) was the average of data, others total terms were the summation of data.

a Correction or removal of erroneous (dirty) data caused by contradictions, disparities, keying mistakes, missing bits, etc.

b Percent of target genome size in the reference genome size.

c Percent of clean data in the reference genome size.

d Percent of bases mapping in the reference genome size.

e Single nucleotide polymorphism.

f Insert and deletion.

g Structural variant.

### SNPs detection

**Figure 3:**
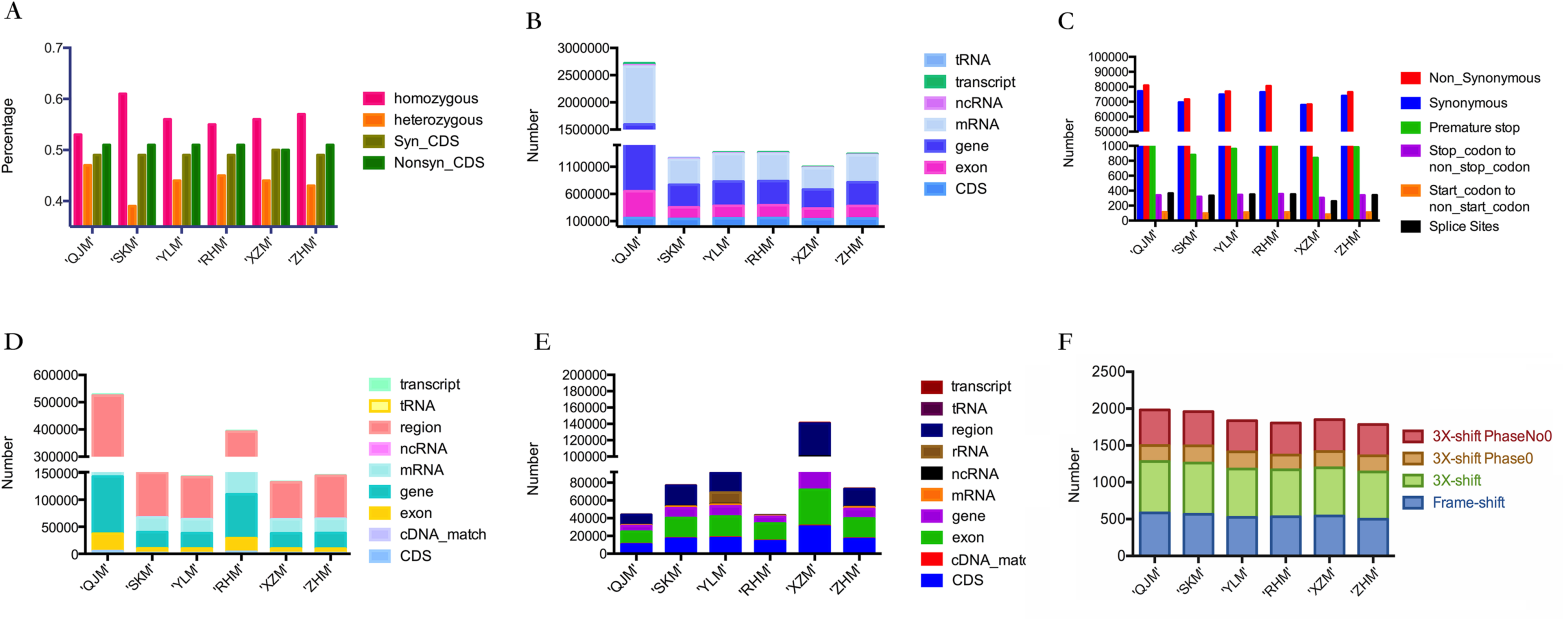
Statistics of SNPs, InDel and SVs. (A) Total number of variants, type and zygosity of variants in each genotype. Syn_CDS means synonymous SNPs in the CDS region, nonsyn_CDS means non-synonymous SNPs in the CDS region; (B) the distribution of SNPs in different regions among all six cultivars; (C) summary of large effect SNPs in all cultivars of Japanese apricot; (D) the distribution of InDels in different regions among all six cultivars; (E) the distribution of SVs in different regions among all six cultivars; (F) frameshift mutation occurred among all Japanese apricot cultivars. Frame-shift means frameshift mutation in the CDS region, 3X-shift means 3X-shift mutation in CDS region, 3X-shift Phase0 means 3X-shift mutation in CDS region (phase 0), 3X-shift PhaseNo0 means 3X-shift mutation in CDS region (phase No 0).

**Figure 4:**
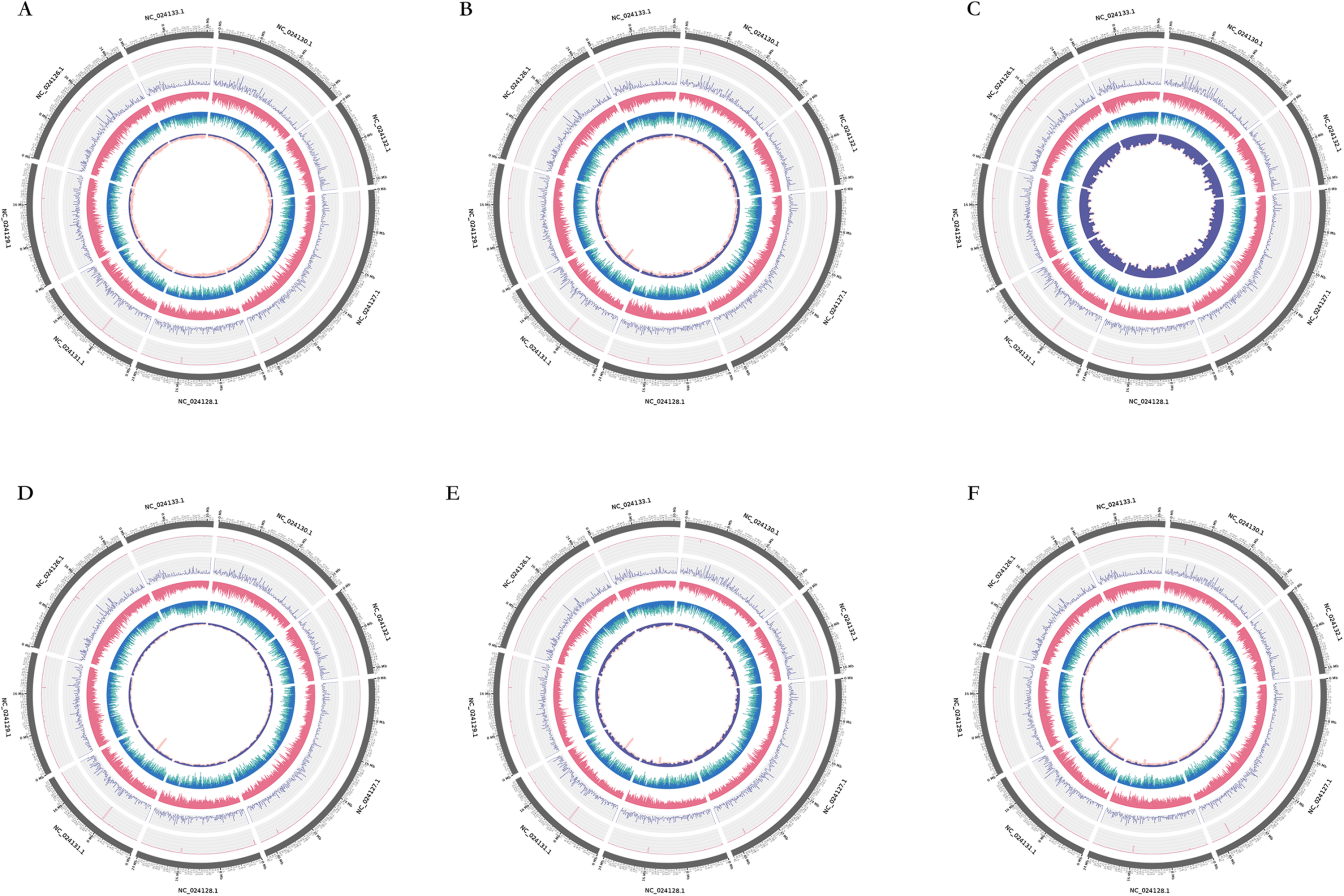
Diagrammatic representation of structural variation in the sequence data obtained from the re-sequencing of six Japanese apricot cultivars. (A) ‘QJM,’ (B) ‘SKM,’ (C) ‘YLM,’ (D) ‘RHM,’ (E) ‘XZM,’ (F) ‘ZHM.’ There are six circles inside and out, and the eight chromosomes are portrayed along the perimeter of the outer circle. The depth of re-sequencing and coverage are represented in the second and third circle, respectively, every window’s size is 100 kb. The forth circle represents the distribution of SNP density (red); the quantity of InDel which contains an insertion (green) or deletion (blue) represents in the fifth circle. The innermost circle indicates the account of SV which contains an insertion (pink) or deletion (purple).

SOAPsnp (http://soap.genomics.org.cn/soapsnp.html) software was used to detect SNPs and considered the main variant among these six genotypes. On the basis of same sequence information (i.e. after comparing all SNPs of the six samples), filtering out the polymorphic loci detected between reference sequence and genotype by low quality and depth, high reliability of SNP data was attained. A total of 1,387,518 SNPs were detected in ‘QJM,’ of which 53.09% were homozygous and 46.91% were heterozygous (Fig. 3A). Approximately 48.90% were synonymous SNPs in CDS regions (Syn_CDS) and 51.10% were non-synonymous SNPs in CDS regions (Nonsyn_CDS). A total of 1,240,360 SNPs were detected in ‘SKM,’ of which 60.54% were homozygous and 39.46% were heterozygous. Approximately 49.41% were Syn_CDS and 50.59% were Nonsyn_CDS. A total of 1,331,341 SNPs were identified in ‘YLM,’ of which 56.42% were homozygous and 43.58% were heterozygous. Approximately 49.46% were Syn_CDS, and 50.54% were Nonsyn_CDS. A total of 1,231,625 SNPs were detected in ‘RHM,’ of which 54.98% were homozygous and 45.02% were heterozygous. Approximately 48.78% were Syn_CDS and 51.22% were Nonsyn_CDS. A total of 894,302 SNPs were detected in ‘XZM,’ of which 56.24% were homozygous and 43.76% were heterozygous. Approximately 49.97% of which were Syn_CDS and 50.03% were Nonsyn_CDS. A total of 1,301,911 SNPs were detected in ‘ZHM,’ of which 56.65% were homozygous and 43.35% were heterozygous. Approximately 49.29% were Syn_CDS and 50.71% were Nonsyn_CDS (Fig. 3A). The distribution of SNPs corresponding to functional element regions (such as CDS, exons, UTR, etc.) revealed that the mRNA region (46.15%) occupied the majority of SNPs among all functional regions, followed by the gene region (41.05%). Exons accounted for third most enriched region, and considered to be an important CDS region, the other three regions (ncRNA, transcript and tRNA) had a small part in SNPs variation. The distribution of SNPs in different regions was not significantly different among the six cultivars, except for SNPs in exon, gene and mRNA regions of ‘QJM’ and SNPs in transcript and tRNA regions of ‘SKM’ (Fig. 3B). The distribution of SNPs types was similar among eight chromosomes in all six cultivars (Table S1; Fig. 4). ‘QJM’ exhibited the highest sequence variation of all six cultivars with respect to the *P. mume* reference genome, while ‘SKM’ showed the lowest sequence variation from all cultivars. The genome-wide mutation rate for ‘QJM’ was one change per 64 bases versus one change per 110 bases for ‘SKM.’ Among eight chromosomes that contain the reference genome, uppermost quantity of SNPs was perceived in chr 2 in all six genotypes. Particularly, chr 7 in both ‘QJM’ and ‘SKM’ showed the lowest level of mutations. Chr 8 in the other four cultivars exhibited the lowest level of mutations, respectively.

Single nucleotide polymorphisms in CDS region are worthy of attention based on explanation of the *P. mume* reference genome. SNPs that change the translation start–stop site or variable splice sites would cause phenotypic change. Approximately 10,397 (0.14%) of identified SNPs were considered large-effect SNPs consisting of premature stop (5,787 SNPs), stop-codon to non-stop codon (1,997 SNPs), start-codon to non-start codon (616 SNPs) and splice site (1,997 SNPs). The cultivar ‘QJM’ demonstrated the highest number of large-effect SNPs (1,882 SNPs), ‘RHM’ (1,878 SNPs) showed no significant difference from ‘QJM,’ followed by ‘ZHM’ (1,768 SNPs), ‘YLM’ (1,762 SNPs) and ‘SKM’ (1,626 SNPs), whereas ‘XZM’ revealed the lowest number of large-effect SNPs (Fig. 3C).

### Short InDels detection

SOAPindel software was used to detect short InDels, in accordance with the statistics of SNPs (Table 4). A total of 1,493,577 InDels were acquired in all six cultivars, and majority of variants were found in region (53.1%), followed by the gene region (20.1%) and mRNA region (18.5%). Approximately 1/3 of all InDels were fixed in ‘QJM’ (527,815), giving ‘QJM’ the highest number of InDels variations. ‘XZM’ contained the lowest number of InDels in all six cultivars (132,876), and remaining four cultivars: ‘SKM’ (150691), ‘YLM’ (142748), ‘XZM’ (132876) and ‘ZHM’ (145182) are shown (Fig. 3D).

The distribution of InDels was not significantly different from that of SNPs among eight chromosomes in all six cultivars. ‘SKM’ (82072) showed highest number of InDels of all six genotypes, followed by ‘ZHM’ (79160), ‘QJM’ (77390) and ‘YLM’ (77381), while ‘XZM’ contained the lowest number of InDels of all cultivars. The percentages of insertions and deletions among eight chromosomes in all six cultivars were similar: insertions ranged from 47.1% to 49.5%, slightly less than deletions (50.5% to 52.9%). Chr 2 had the most abundant variation of all eight chromosomes, accounting for 20% of all variation (Table S2).

**Figure 5:**
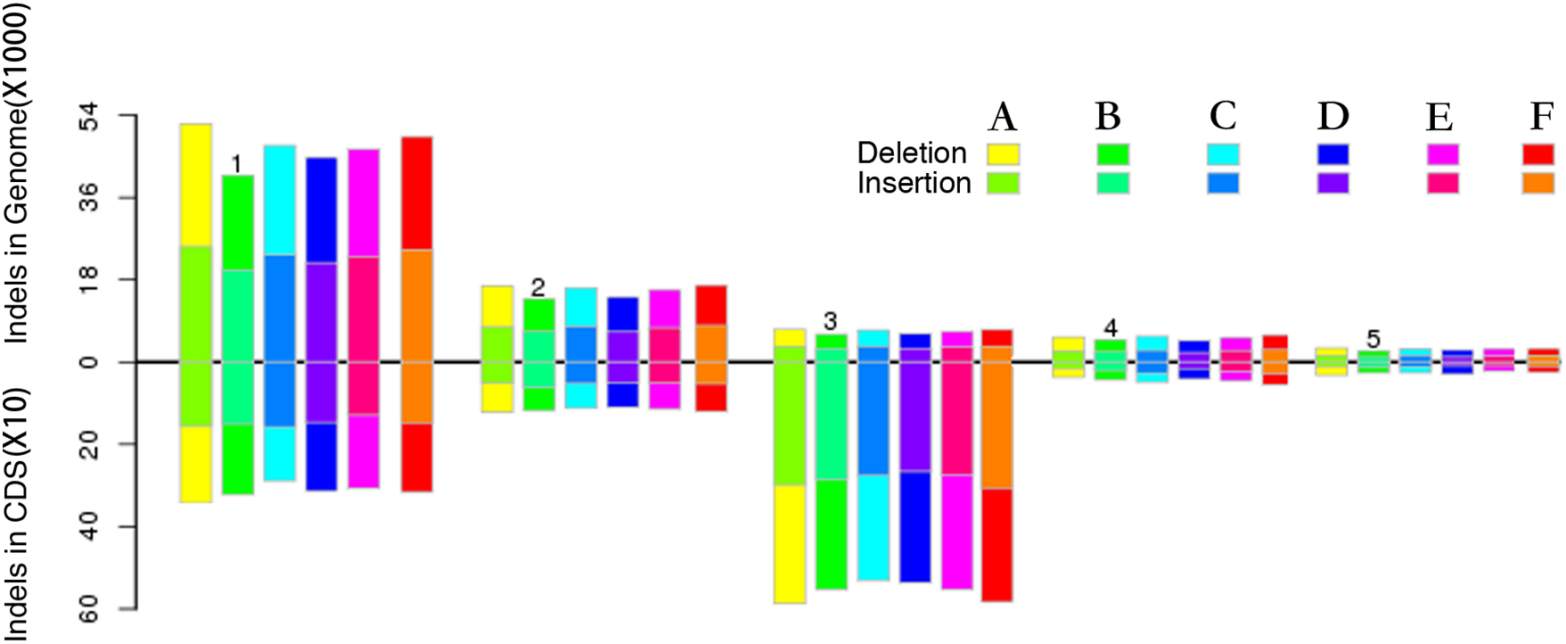
Different InDel size distributions and frameshift mutation s between the whole genome and CDS region. (A) ‘QJM,’ (B) ‘SKM,’ (C) ‘YLM,’ (D) ‘RHM,’ (E) ‘XZM,’ (F) ‘ZHM.’ The *y*-axis indicates the number of InDels in the CDS and genome, the *x*-axis indicates the different sizes of InDel distributions and frameshift mutations.

Frameshift mutations are genetic mutations affected by insertions and deletions of multiple nucleotides in a DNA sequence. Some InDels were projected to be frameshift mutations, but the lengths of InDels in coding regions were more probable from 1 to 5 (the length of a codon), than InDels in the rest of the genome (Fig. 5). In this study, two types of mutation were detected; the most frequent frameshift was a 3X-shift mutation in CDSs (3987), including phase0 (1,320) and phaseNo0 (2,667), followed by frameshift mutations in CDSs, which were the second highest number of mutations (3,245), whereas ‘QJM’ had the highest number of two-shift mutations (Fig. 3F).

### SVs detection

Structure variation was important between individuals of the same species, and the types detected could be insertions and deletions, repetitions, inversions and translocations. Because of their small contribution to variation, types of variation other than insertions and deletions were classified as others. The distribution of SVs in different regions showed (Fig. 3E) no significant difference between SNPs and InDels, and this region was the highest amount of structure variation among all regions. ‘XZM’ had the most variation in CDSs, the regions of most interest to us, ‘QJM’ cultivar has the least variable while other four cultivars were not significantly different. The distribution of SVs was not significantly different from that of InDels and SNPs among eight chromosomes in all six cultivars (Table S3). The highest variation was observed on chr 2 (16.6%–18.4%), and distribution of other chromosomes was similar. The percentage of insertions and deletions was significantly different among all cultivars between green- and red-skinned: approximately 60% of SVs was insertion in ‘QJM,’ while ‘YLM’ had the lowest number of insertions of all cultivars (7%). A total of 40,055 SVs were detected in ‘QJM,’ in which insertions accounted for 60% (the highest amount), and deletions accounted for 43.76% (the lowest amount) of the identified SVs. The opposite trend was observed in ‘YLM’: it had the lowest number of insertions and the highest number of deletions, whereas the distributions of other types of SVs differed little, one to another.

### Functional annotation of databases

**Figure 6:**
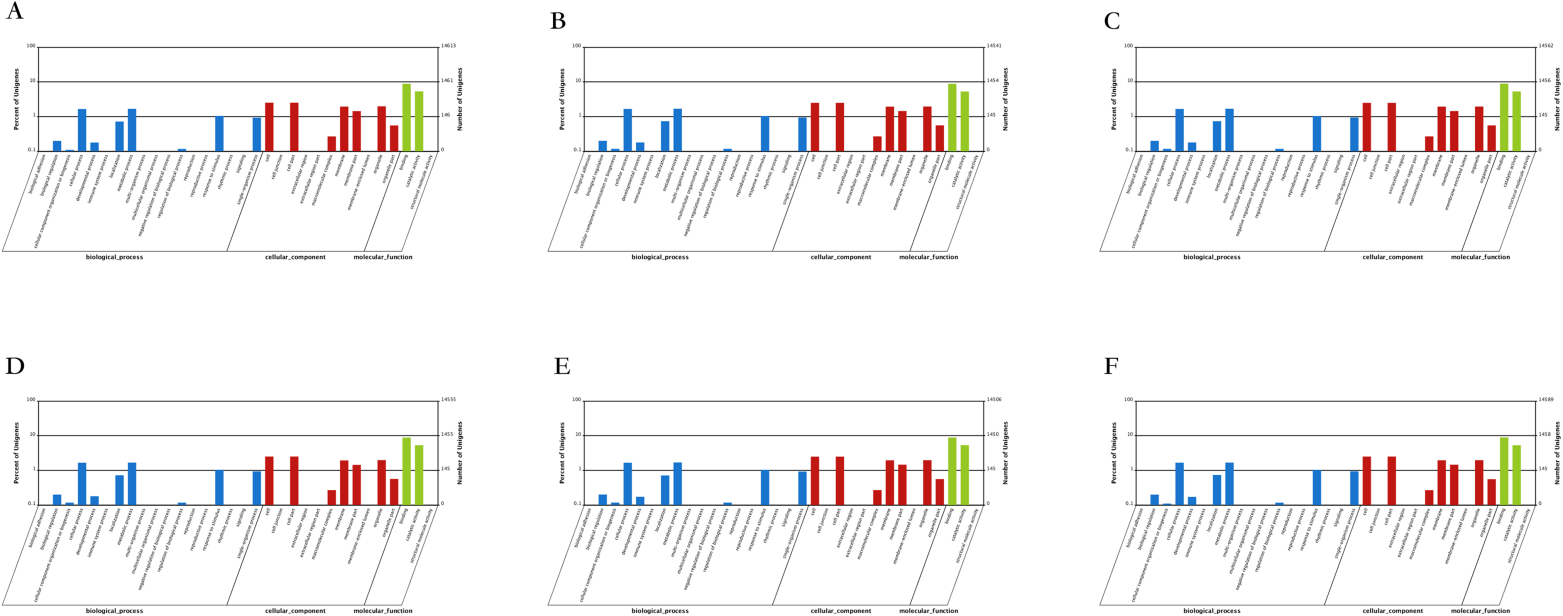
GO classification for differential unigenes between the six Japanese apricot cultivars. The results are the percentage of each GO category of genes in groups of cellular component, molecular function and biological process. The left *y*-axis indicates the percentage of genes in a category, and the right *y*-axis indicates the number of genes in a category. The *x*-axis indicates the three ontologies, respectively. A corrected (*p* ≤ 0.05) was chosen as the threshold value. The GO term (*p* ≤ 0.05) is defined as a differentially expressed gene-enriched GO term.

A change in function of a gene was usually because of the existence of non-synonymous (NS) mutation, frameshift (F) and SVs; in turn, expression of related protein was influenced. All tested and functionally explained genes were categorized into GO (Fig. 6). Approximately 4,736 genes per cultivar were separated into three groups: molecular function, cellular components and biological process. Among all categories, the highest number of genes (2,060 on average) were recorded in molecular function, while the lowest number of genes were observed in cellular components (1,657) and biological process categories (1,020), respectively. GO enrichment classification recommended that the genes from molecular function, cellular component and biological process groupings could be classified into three, 11 and 18 groups, respectively.

**Figure 7:**
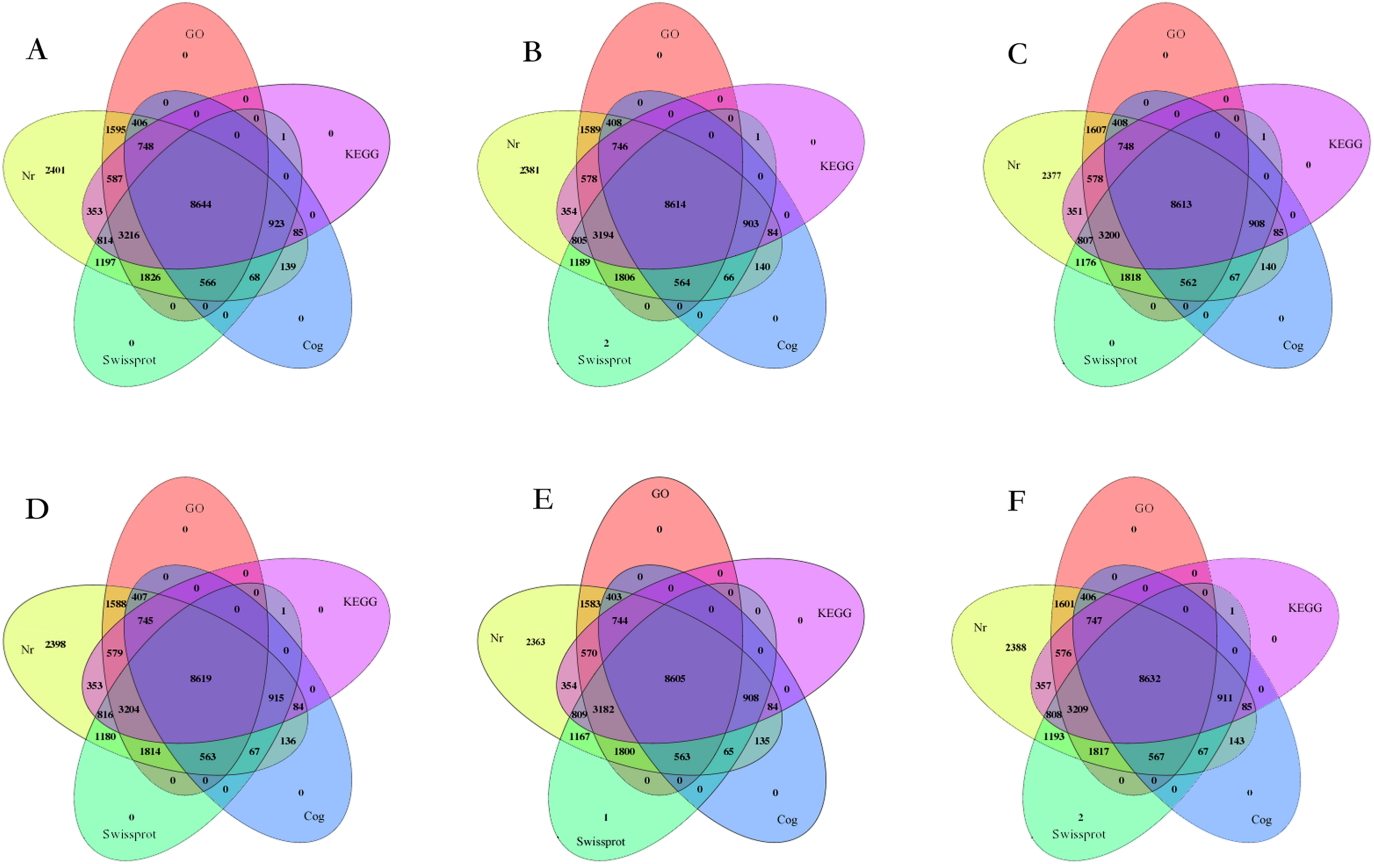
Homology analysis of the six Japanese apricot cultivars relative to reference genome. (A) ‘QJM,’ (B) ‘SKM,’ (C) ‘YLM,’ (D) ‘RHM,’ (E) ‘XZM,’ (F) ‘ZHM.’ A total of six public databases included NR—Non-redundant sequence databases, SwissProt—Swiss-Prot protein database, GO—Gene Ontology, COG—Clusters of Orthologous Groups and KEGG—Kyoto Encyclopedia of Genes and Genomes.

Genes explained by BLASTx, with a threshold of 10^−5^, were noted in six public databases comprising the GO, NCBI non-redundant (NR), nucleotide sequence (NT), Swiss-Prot, Clusters of Orthologous Groups of Proteins (COG) and KEGG databases. A total of 23,698 genes in ‘QJM,’ 23,554 genes in ‘SKM,’ 23,572 genes in ‘YLM,’ 23,597 genes in ‘RHM,’ 23,458 genes in ‘XZM’ and 23,640 genes in ‘ZHM’ were annotated successfully (Fig. 7). All the genes were noted in the NR database, and the distributions of genes annotated in six databases were not significantly different. At least 8,605 genes (‘XZM’) were annotated in all six databases, while ‘QJM’ had the most genes annotated in all databases. The other four cultivars showed 8,614 genes in ‘SKM,’ 8,613 genes in ‘YLM,’ 8,619 genes in ‘RHM’ and 8,632 genes in ‘ZHM.’

Overall, CDS is a region of DNA that is translated to form protein, and detection of sequence variation occurring in the CDS region becomes extremely important for gene function analysis. Through genome-wide comparative analysis of six re-sequence data sets in the CDS region, a total of 181,331 positions of SNPs, 3,318 positions of InDels and 51,427 SVs detected 19,774, 1,937 and 13,850 unigenes, respectively. The significant difference between green- and red-skinned cultivars is the colour pigmentation due to existence of anthocyanin, in order to obtain the same sequence variations occurring in all green-skinned cultivars, while red-skinned cultivars have either no variation or synonymous mutations only associated with the biosynthesis and accumulation of anthocyanin. After comparison found that a sum of 3,143 SNPs (1,350 unigenes), 60 InDels (32 unigenes) and 337 SVs (122 unigenes) differed between green- and red-skinned cultivars (Table 5), including 25 unigenes containing two sequence variations at same time, it was a simple task to find the important variations which leads to pigmentation changes: there are four pathways, namely phenylpropanoid biosynthesis (31 unigenes, ko00940), flavonoid biosynthesis (15 unigenes, ko00941), flavone and flavonol biosynthesis (seven unigenes, ko00944), isoflavonoid biosynthesis (two unigenes, ko00943), that were found to be related to biosynthesis of anthocyanin according to previous research results. TFs (26 unigenes), as a series of regulatory genes that control structural gene transcription, were detected, including genes encoding MYB (four unigenes), bHLH (two unigenes), WD40-repeat protein (one unigenes), NAC (one unigenes), etc. Twenty-one unigenes may be related to accumulation of anthocyanin.

**Table 5:** Candidate genes participated in the biosynthesis of anthocyanin.

	SNP	InDel	SV
All positions	181331	3318	51427
All unigenes	19774	1937	13850
Compare positions	3143	60	337
Compare unigenes	1350	32	122

### Genes validation by qRT-PCR at transcriptional level

**Figure 8:**
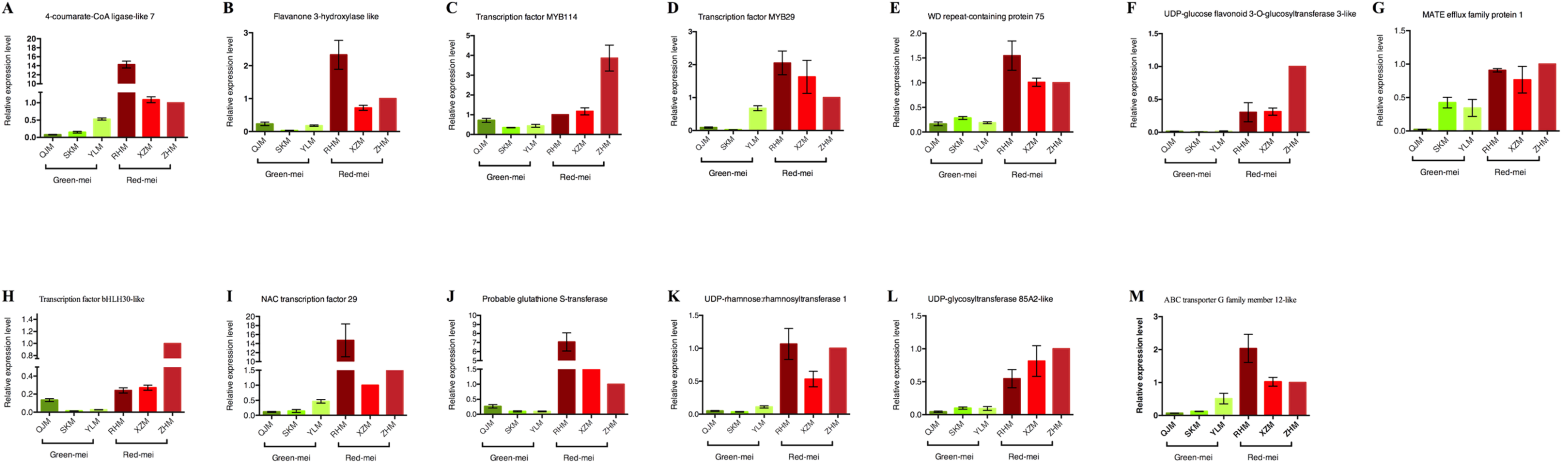
Expression analysis of the anthocyanin biosynthetic genes in all of Japanese apricot cultivars using qRT-PCR. Relative levels of gene expression were calculated using RP II. The vertical bars represent the standard deviation (SD) of three biological replicates.

Fruit colour is determined by the accumulation and biosynthesis of anthocyanins, mediated by multiple enzymes in the phenylpropanoid and flavonoid pathways, regulatory genes and TFs. According to functional annotation results, 13 candidate homologous genes were revealed related to colour pigmentation, the translated proteins were detected as NS, InDels or SVs and 13 candidate genes participated in the biosynthesis of anthocyanin (Table 6). The expression patterns of genes encoding three anthocyanin synthesis structural genes (*4CL*, *F3H* and *UFGT*), five TFs (MBW complexes and NAC) and five related regulated genes (*GST1*, *RT1*, *UGT85A2*, ABC and MATE transports) were definite through quantitative reverse transcription-polymerase chain reaction (qRT-PCR), expression pattern of these candidate genes were not significantly different, and transcription levels of genes in red-skinned cultivars were significantly higher than those of the genes in green-skinned cultivars (Fig. 8).

**Table 6:** Candidate genes associated with the anthocyanin synthesis.

Functions	Description	Gene	Type red-skinned	Type green-skinned
Structural genes	4-Coumarate-CoA ligase-like 7	4-CL	S^a^	NS^b^/InDel^c^/SV^d^
	Flavanone 3-hydroxylase	F3H	S	NS
	UDP-glucose flavonoid 3-*O*-glucosyltransferase 3-like	UFGT	S	NS
Transcription factors	Transcription factor MYB114	MYB114	S	NS
	Transcription factor MYB29	MYB29	NS	S
	WD repeat-containing protein 75	WD40	S	S/NS
	Transcription factor bHLH30-like	bHLH30	NS	S
	NAC transcription factor 29	NAC	NS	S
Related genes	Probable glutathione S-transferase	GST1	S	NS
	UDP-rhamnose: rhamnosyltransferase 1	RT1	NS	S
	UDP-glycosyltransferase 85A2-like	UGT85A2	NS/SV	S
	ABC transporter G family member 12-like	ABCG12	S	NS
	MATE efflux family protein 1	MATE1	S	NS

**Notes:**

a There are synonymous SNPs in genes.

b There are non-synonymous SNPs in genes.

c There are insert or deletion in genes.

d There are structure variation in genes.

### UFGT enzyme analysis

**Figure 9:**
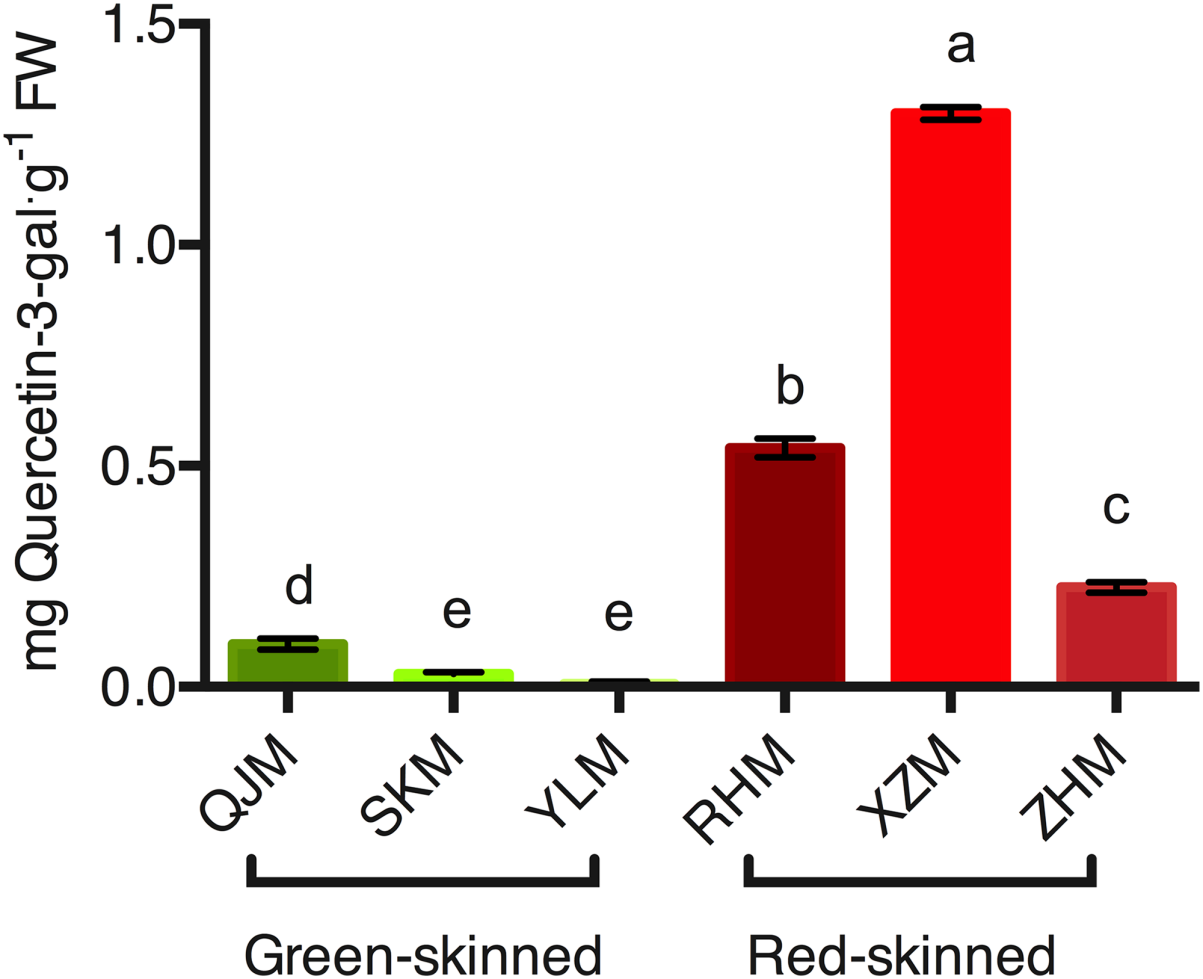
Assay of UFGT activity between the six Japanese apricot cultivars. Values represent mean ± standard deviation, *n* = 3. Different letters in rows indicate significant differences among mean values of treatments (*p* < 0.005).

UDP-glucose flavonoid-3-*O*-glycosyltransferase is assumed to be an important enzyme for anthocyanin biosynthesis in many plants. In this study, UFGT activity was identified in all cultivars. While the lowest activity was 0.01 mg g^−1^ in ‘YLM’ and 0.03 mg/g in ‘SKM,’ compared with green-skinned cultivars, red-skinned cultivars showed significantly higher UFGT activity. ‘XZM’ cultivars had the highest activity (1.3 mg/g) of all cultivars, followed by ‘RHM’ (0.5 mg/g) and ‘ZHM’ (0.2 mg/g) (Fig. 9).

## Discussion

In the present study, we mainly focused on understanding the colouring mechanism in Japanese apricot. Different experiments were conducted for this study. Firstly, we used UPLC/MS method to identify the main anthocyanin components, we identified a component ‘cyanidin-3-glucoside’ a major anthocyanin in Japanese apricot fruit, consistent with previous studies conducted in flowers (Changling, Weiming & Junyu, 2004). However, the molecular mechanisms of anthocyanin biosynthesis in Japanese apricot are not yet clear. After that, we performed genome re-sequencing to investigate the molecular mechanism of synthesis and accumulation of anthocyanin, comparing gene variations between red- and green-skinned cultivars and confirmed using quantitative qRT-PCR to find the expression pattern of those candidate genes, we found that three anthocyanin synthesis structural genes (*4CL*, *F3H* and *UFGT*), five TFs (MBW complexes and NAC) and five genes (*GST1*, *RT1*, *UGT85A2*, ABC and MATE transports) were possibly involved in the anthocyanin metabolic pathway of Japanese apricot. In addition, developing SNP makers in a wider range of Japanese apricot varieties by using re-sequencing data.

### Genes encoding enzymes in the anthocyanin biosynthetic pathway

Multiple enzymes participated in anthocyanin biosynthesis in phenylpropanoid and flavonoid pathways. In this study, three candidate enzymes were discussed (Fig. 8). 4CL was the final enzyme of the general phenylpropanoid pathway, converting phenylalanine to 4-coumaroyl-CoA, a product that resulted in a range of flavonoid compounds, such as pro-anthocyanidins and anthocyanins (Boss, Davies & Robinson, 1996b). Ozeki (Ozeki & Komamine, 1985) studied the regulated relationship between 2,4-D and anthocyanin synthesis, and when no anthocyanin synthesis occurred, the activities of 4CL increased one day after transfer due to transfer effect but consequently decreased and remained at low levels. When the activities of 4CL increased, and reached up to maximum, anthocyanin was synthesized most rapidly. Christie (Christie, Alfenito & Walbot, 1994) found anthocyanin accumulation in all tested lines with 4CL-homologous transcripts increased at least threefold over levels in unstressed plants (no anthocyanin accumulation). These observations agree well with those qRT-PCR results mentioned above.

The primary phases of the flavonoid pathway were from 4-coumaroyl CoA through chalcone and naringenin to dihydroflavonol, in which F3H was one of three main enzymes (other two were CHS, CHI converted naringenin flavanone to dihydroflavonols). *F3H* gene expression seems to be fundamental in regulation at bifurcation of anthocyanin and flavonol branches, catalyses the stereospecific hydroxylation of (2S)-flavanones at 3-position of C-ring to (2R, 3R)-dihydroflavonols (Sparvoli et al., 1994). Zuker (Zuker et al., 2002), working on antisense suppression to block the expression of a flower colour gene encoding *F3H* of carnation (*Dianthus caryophyllus* L.), found that transgenic plants showed flower colour alterations ranging from attenuation to complete loss of their orange/reddish colour. *F3H* expression was explored to clarify the molecular mechanism of red colouration in apple; results indicated that the *F3H* gene was co-ordinately expressed during fruit development, and its levels of expression was positively related to the degree of anthocyanin concentration (Honda et al., 2002). Castellarin (Castellarin et al., 2007) performed dehydration to magnify the expression of *F3H* gene, which described well the increase of anthocyanin content in grapevines (*Vitis vinifera* L.) during experimental seasons. In radish (*Raphanus sativus*) varieties, the *F3H* gene was remarkably expressed through accumulation of sucrose in red radish; in contrast, the expression of *F3H* was powerfully suppressed in the white variety despite the accumulation of sucrose, showing that *F3H* activated the biosynthesis of anthocyanins via regulation of TFs (Hara et al., 2004).

UDP-glucose flavonoid-3-*O*-glycosyltransferase, an enzyme participated in the late step in anthocyanin biosynthesis, transfers the glucosyl moiety from UDP-glucose to the 3-hydroxyl group of acceptor molecules in a glucosyltransferase catalytic reaction. The role of UFGT is to anthocyanidins were glucosylated by UFGT during red-skinned fruit ripening. Dissimilarities in colour strength make contribution to the expression differences of structural genes. High expression of UFGT has been detected in red skin of grapes, while in white grapes, almost extremely low expression was found (Boss, Davies & Robinson, 1996b). The lack of anthocyanins detected in white Malay apple fruits was due to the absence of detectable levels of UFGT transcripts (Kotepong, Ketsa & van Doorn, 2011). Takos (Takos et al., 2006) confirmed that the transcript levels of genes encoding UFGT in multiple anthocyanin pathways was much higher in red-skinned apples than in others. The transcript expression pattern of gene encoding UFGT reach the fastest accumulation speed at both primary and late developmental stage was confirmed in other fruits like peach (Tsuda et al., 2004), bilberry (Jaakola et al., 2002).

In the current study, transcript levels of 4CL, F3H and UFGT, consistent to the flavanol and anthocyanin levels, were greater in red-skinned fruits than in green-skinned cultivars (Fig. 8); up-regulated structural genes in red-skinned fruits indicated three genes contributed in the accumulation of anthocyanin that led to red pigmentation in plants.

### Regulation of TFs through anthocyanin accumulation

The anthocyanin biosynthetic pathway has been comprehensively studied in several species such as tobacco (Aharoni et al., 2001), *Arabidopsis* (Tohge et al., 2005), grapevine (Deluc et al., 2006), apple (Espley et al., 2007), tomato (Butelli et al., 2008), mangosteen (Palapol et al., 2009) and Chinese bayberry (Niu et al., 2010); the structural genes encoding pathway enzymes have been mentioned above, and other regulatory genes were TFs which could control structural gene transcription (Fig. 8).

The anthocyanin pathway genes are regulated by interaction of DNA binding R2R3-MYB TFs and MYC-like bHLH and WD40-repeat proteins (MBW complexes) in plants (Chagné et al., 2007). Two MYB genes, *MYB29* and *MYB114*, were found in this present study to be pertain in regulation of anthocyanin synthesis (Li et al., 2014; Schwinn et al., 2016; Sun et al., 2016) (Fig. 8). Schwinn (Schwinn et al., 2016) reported that *MYB29* was associated with bulb colour (*Allium cepa* L., Allioideae, Asparagales): as an R2R3-MYB TF that regulated the flavonoid pathway, it was demonstrated that *MYB29* belonged to sub-group that carried out biosynthesis of flavonoids. Overexpression of *MYB5* or *MYB114* strongly induces proanthocyanidin (PA) accumulation in *Medicago truncatula* hairy roots, and both *MYB5* and *MYB114*, mutants of *M. truncatula,* which exhibit darker seed coat colour than wild-type plants, were revealed to physically interact and synergistically activate the promoters of anthocyanidin reductase and leucoanthocyanidin reductase (Liu, Jun & Dixon, 2014). Gonzalez (Gonzalez et al., 2008) reported that overexpression of *MYB114* resulted in enhanced pigment production in a TTG1- and bHLH-dependent manner. Li (Li et al., 2014) found that JA promotion of anthocyanin accumulation under far-red light is dependent on the phytochrome A signalling pathway; at the same time, knockdown expression of *MYB114* significantly reduces methyl jasmonic acid promotion of anthocyanin accumulation.

As a regulatory complex comprising another candidate TF, bHLH30-like (bHLH30), and WD repeat-containing protein 75 (WD40), the expression arrangements of these two candidate genes, *bHLH30* and *WD40*, in six cultivars were the same as those of MYB family genes (*MYB5* and *MYB114*), which are upregulated throughout fruit ripening in red-skinned associated with green-skinned cultivars. Lc (leaf color) as the first plant bHLH protein involved in maize tissue-specific anthocyanin pigmentation by mutant analyses (Dias et al., 2003). Zhou (Zhou, Shi & Xie, 2012) reported that nitrogen regulated the synthesis of anthocyanin in red cells operates through the mechanism by which the expression levels of genes encoding equally major constituents of *TTG1*–*GL3*/*TT8*–*PAP1* complex and negative regulators was effected by nitrogen, which was a complex of bHLH and WD40; this may be same as in our present study. All three regulatory complex genes were highly upregulated in red-skinned relative to green-skinned cultivars.

Like MBW regulatory complex, the TF NAC was also found and related to red colour formation. The NAC family are plant-particular TFs and have 106 and 149 members pretend in *Arabidopsis* and rice, correspondingly (Gong et al., 2004; Xiong et al., 2005). Morishita (Morishita et al., 2009) found that NAC078 protein regulated the expression of genes related to the biosynthesis of flavonoids and the level of anthocyanins were expressively increased in NAC078-containing plants and reduced in NAC078-knockout plants of *Arabidopsis*. In orange fruit, a gene encoding TF NAC domain protein was induced the biosynthesis of anthocyanins in blood oranges but not in common oranges. The same phenomena occurred in raspberry in a previous study: a candidate gene encoding NAC (*CUC2-like*) was accountable for the accumulation of cyanidin and pelargonidin pigments (Kassim et al., 2009). Our study showed that NAC TF 29 was highly upregulated in red-skinned relative to green-skinned cultivars, which suggested that it is a potential TF contributing to the regulation of red colour formation in Japanese apricot.

Among these candidate TFs, the functions of only two MYB TFs (MYB29 and MYB114) have been studied in *Arabidopsis* and other plants, whereas reports on TF function are rare in Japanese apricot (Fig. 8). The other three candidate genes have not been stated to be intricate in the biosynthesis of anthocyanins. Advance studies are desired to regulate whether changes in the transcription of these candidate genes are correlated to regulation of anthocyanin metabolism. Moreover, in Japanese apricot, the relationship between regulatory complex (MBW) and anthocyanin biosynthesis leftovers is unclear. Subsequently, this matter should also be explored.

### Related genes contributed to accumulation of anthocyanin

There were several candidate genes participating in biosynthesis or the accumulation of anthocyanin. However, these genes were not the same as structural genes, which could directly impact the anthocyanin synthesis pathway, and neither were they like TFs, which could control structural gene transcription; they may be related to regulation of the anthocyanin biosynthesis pathway.

In this study, seven candidate genes were identified that might be associated to the biosynthesis of anthocyanin (Fig. 8). Glutathione S-transferase (*GST1*) probably have the same function as *Bz2* gene in maize (Marrs et al., 1995), which encodes a protein with GST activity conjugated with glutathione and the inhibitor vanadate in plant tonoplast and has been shown to inhibit the accumulation of anthocyanins in vacuole. Alfenito (Alfenito et al., 1998) revealed that *An9*, which encoded a kind of glutathione S-transferase which transported glutathionated cyanidin 3-glucoside (C3G) to the vacuole, regulated transcriptional activator of the anthocyanin pathway. In *Vitis vinifera* L., two other GST genes (*VvGST1* and *VvGST4*) showed different induction patterns, but all their transcriptional profiling showed that they induced intensively different accumulation of anthocyanin from that of *Bz2* and *An9* in maize and petunia, correspondingly (Conn et al., 2008). TT19 was demonstrated in *Arabidopsis* that works as a carrier for uptake of anthocyanins or proanthocyanidin precursors into the vacuole and may protect cyanidin from degradation during transport process (Kitamura, Shikazono & Tanaka, 2004). GST in strawberry was also mentioned as one of the list of differentially expressed gene related to biosynthesis anthocyanin (Gu et al., 2015).

In *petunia hybrida,* expression of UDP-rhamnose/rhamnosyltransferse (Rt) gene was induced by intense light, and transcription of UDP-rhamnose: anthocyanidin-3-glucisde rhamnosyltransferse (3RT) played a key role in promoting the accumulation of anthocyanin in epidermal cells of the petal (Morita et al., 2005). Regarding secondary metabolism in Norton grape (Ali et al., 2011), the transcriptional profile of UDP-rhamnose/rhamnosyltransferse was proposed to be associated with the anthocyanin pathway. The RT gene downregulated the activity of *PAL* when it was low; however, *PAL* was a structural gene in the anthocyanin pathway, indicating that RT gene influenced the biosynthesis of anthocyanin (Given, Venis & Grierson, 1988).

The transcriptional expression of UDP-glycosyltransferase 85A2*-*like (UGT85A2) was highly upregulated in red-skinned comparative to green-skinned cultivars, indicating that it may be associated with the biosynthesis of anthocyanin. In Crocus species, transcription of UDP-glycosyltransferase 703B1 was found only in stigmas and petals where anthocyanin accumulates (Nørbæk et al., 2002; Moraga et al., 2009). A relationship between *Arabidopsis thaliana* mutant UGT72B1 and decreased accumulation of anthocyanin in floral stems was confirmed, and an association between UGT86A1 with the anthocyanin biosynthesis pathway in strawberry was also mentioned (Gu et al., 2015). The ABC and MATE transporter families are two important groups of proteins whose members participate in an extensive variety of transport processes. The upregulation of ABC transporters demonstrated that ABC transporters take part in transport of anthocyanin into vacuoles of *Arabidopsis* (Lu, Li & Rea, 1997). In yeast, two multidrug resistance-associated protein-type ABC transporters were found to facilitate vacuolar uptake of anthocyanin–glutathione conjugates (Rea, 1999), and same results were reported in maize (Debeaujon et al., 2001) and tea (Li, 2016). The MATE transporters TT12 of *Arabidopsis* work as a precursors that active in cells synthesizing proanthocyanidins, thus inducing vacuolar accumulation of proanthocyanidins in the seed (Gomez et al., 2009).

All related genes intricate in regulation of the biosynthesis of anthocyanin were studied in this experiment and showed same expression pattern as the structural genes and TFs, also representing that these genes may be associated to anthocyanin accumulation.

### Gene expression encoding UFGT enzyme

As the gene encoding enzyme accountable for preceding step in biosynthesis of anthocyanin, the *UFGT* gene played an important role in preeminent substances of anthocyanins and flavonols in red pericarps and their effective absence in green pericarps. In this study, gene expression of *UFGT* was significantly upregulated in red pericarps, while almost no one was detected in green pericarps (Fig. 9). At the same time, the enzyme activity was detected among all Japanese apricot cultivars, whether in coloured skin where anthocyanin was accumulated or in green pericarps where no colour pigmentation was present, but the activity of UFGT was expressively greater in red pericarps than in green pericarps (Fig. 9). Studies of other plant species have shown that the expression of *UFGT* gene was acute for anthocyanin biosynthesis. For example, the expression of *StUFGT*, which induced corresponding by gibberellic acid and sucrose, was detected in roots, leaves and stems, suggested that UFGT was related to the accumulation of anthocyanin in potato (Hu et al., 2011). In grape berry, *UFGT* gene expression was only identified in red-skinned spots, and in Kyoho it was detected by northern blot analysis, indicating that the *UFGT* gene is a main regulator of anthocyanin biosynthesis (Kobayashi et al., 2001). Unlike other genes of the anthocyanin pathway, expression of UFGT was expected to mirror the anthocyanin content, suggesting that fruit colouration was strongly influenced by UFGT expression in litchi (Zhao et al., 2012). In conclusion, the *UFGT* gene was the most important gene correlated with the biosynthesis and accumulation of anthocyanins, contributing to red pigmentation of Japanese apricot.

## Conclusion

Empiric genome re-sequencing on an Illumina platform was a realistic means to scientifically investigate the universal genomic variations associated with the accumulation of anthocyanins in Japanese apricot fruits. We investigated that the genes participating in developments of anthocyanin biosynthesis, TFs and related genes were only upregulated in red-skinned cultivars, confirming that these genes may have the function of regulating anthocyanin biosynthesis. Generally, colourful fruits such as those of the ornamental Japanese apricot are very attractive to consumers. Considering the molecular mechanisms underlying their formation is imperative, predominantly for understanding the transcriptional regulatory networks of dissimilar coloured fruits and alterations in accumulation and distribution of different compounds in coloured cultivars. This data can provide valuable information and will subsidize to the selection of plant germplasm resources for marker-assisted selection as well as for developing the nutritional and medicinal importance of Japanese apricot in the future.

## Supplemental Information

10.7717/peerj.4625/supp-1 Supplemental Information 1 The distribution of SNPs in all eight chromosomes among all six cultivars.

10.7717/peerj.4625/supp-2 Supplemental Information 2 The distribution of InDels in all eight chromosomes among all six cultivars.

10.7717/peerj.4625/supp-3 Supplemental Information 3 The distribution of SVs in all eight chromosomes among all six cultivars.

## Additional Information and Declarations

### Data Availability

The following information was supplied regarding data availability: The raw sequencing data in this paper are uploaded to the GenBank SRA database. The BioProject is PRJNA371370. The data for the ‘Xiaoyezhugan’ cultivar have been deposited at SRA under accession SRR5241555. The data for ‘Zhonghong’ cultivar have been deposited at SRA under accession SRR5241554. The data for the ‘Shinanokoume’ cultivar have been deposited at SRA under accession SRR5241553. The data for the ‘Yanglao’ cultivar have been deposited at SRA under accession SRR5241552. The data for the ‘Ruantiaohongmei’ cultivar have been deposited at SRA under accession SRR5241550. The data for the ‘QingjiaNo.2’ cultivar have been deposited at SRA under accession SRR5241549.
